# Metasurface absorber for millimeter waves: a deep learning-optimized approach for enhancing the isolation of wideband dual-port MIMO antennas

**DOI:** 10.1038/s41598-024-81854-5

**Published:** 2024-12-04

**Authors:** Nagesh Kallollu Narayanaswamy, T. Y. Satheesha, Yazeed Alzahrani, Ashish Pandey, Ajay Kumar Dwivedi, Vivek Singh, Manoj Tolani

**Affiliations:** 1 Department of Electronics and Communication Engineering, Nagarjuna College of Engineering and Technology, Bengaluru, India; 2 School of Computer Science Engineering, REVA University, Bengaluru, Karnataka India; 3 Department of Computer Engineering and Information, College of Engineering, Prince Sattam Bin Abdulaziz University, Wadi Addwasir, Saudi Arabia; 4 Department of Data Science and Engineering, Manipal University Jaipur, Jaipur, Rajasthan India; 5 Department of Information and Communication Technology, Manipal Institute of Technology, Manipal Academy of Higher Education, Manipal, Karnataka India

**Keywords:** Engineering, Electrical and electronic engineering

## Abstract

In this communication, the concept of metasurface absorber is utilized to enhance the isolation in the dual port multiple-input multiple-output (MIMO) antenna specially designed for a wideband millimeter wave operation. The frequency of operation of the designed module is 32.5–42.5 GHz with sufficient gain attributes. The designed metasurface array consists of two circular rings on two different layers. The concept of deep learning is utilized to optimize the dimensional configuration of the metasurface to achieve the maximum absorption of electromagnetic waves in the band of interest. The suppression of mutual coupling by double-ring metasurface is analyzed with the help of the wave theory concept. In contrast to previous decoupling methods using metasurfaces, the suggested metasurface is intended to be in the same plane as the array. The findings demonstrate the capability of effectively separating antenna elements in wideband MIMO antenna without compromising the geometrical complexity. Diversity metrics such as envelope correlation coefficient (ECC), mean effective gain (MEG), channel capacity loss (CCL), and total active reflection coefficient (TARC) for the proposed frequency range are also used to evaluate the performance of the constructed MIMO antenna. The wideband characteristics with a compact configuration make the design MIMO module a suitable candidate for mm-wave applications. The congruence between simulation and measurement confirms the validity of the suggested design.

## Introduction

As the yearly data traffic experiences a significant surge, wireless networks may need to accommodate several times more capacity in the future compared to what is now required. To address the rapidly increasing demand, the fifth generation (5G) systems, capable of achieving peak throughput of several gigabits per second (Gb/s), are seen as a significant answer for future communication applications^1–3^. Academic researchers and engineers are using the mm-wave frequency for 5G wireless communication systems since the sub-6 GHz region has limited bandwidth and channel capacity^4,5^.

The upcoming 5G technology is essential for meeting the increasing demands for high data rates, low power consumption, and reliability in a rapidly growing number of connected devices. Additionally, it enhances the capabilities of emerging technologies such as smart cities, Internet of Vehicles, and virtual reality^6^. In contrast, the deterioration of the signal is amplified at the mm-wave Spectrum due to variables such as air absorptions and route loss attenuation^5^. Due to the paramount importance of frequency band allocation in communication system design, the global telecommunications industry and regulatory authorities are making significant efforts to standardize 5G communication networks. Antennas are essential components for the effective implementation of communication networks. Hence, the design of the antenna has immense importance in achieving communication at the millimeter wave frequencies. The anticipated 5G systems will have a multitude of antennas located at both the base station and user terminals. The arrays, MIMO, and beamforming technologies are crucial factors in enabling 5G mm-wave communication^4^. In recent times, there have been several research studies that have investigated antenna designs for the possible millimeter-wave bands^7–13^.

Since its proposal in 2011, metasurface has been widely used for different functions due to its impressive ability to manipulate wavefronts^14^. These functions include anomalous reflection^15^, focusing^16^, multiple beams^17^, vortex generation^18^, surface plasmon polariton excitation^19^, and radar cross-section reduction^20^. In addition, metasurfaces are commonly used to enhance antenna performance. Some of the main applications include improving gain, expanding bandwidth, enabling dual-frequency/multi-frequency operation, and achieving miniaturization^21–24^.

Metasurface-based decoupling technology is used to solve the problem of mutual coupling among array antenna components, which is a significant obstacle in developing MIMO (multiple-input-multiple-output) antenna arrays. Mutual coupling often occurs when the elements of an array are positioned close to one other, leading to various adverse effects that degrade the effectiveness of the antenna, especially for MIMO arrays. The consequences mentioned include impedance mismatching, radiation pattern deviation, side-lobe level rise, scan blindness, high signal correlation, and poor radiation spectrum efficiency^25^. While mutual coupling is often not considered significant when the separation between elements is more than half of the wavelength, it becomes important due to the growing need for downsizing and restricted space. Various decoupling strategies have been developed to minimize mutual coupling between components of an antenna array. Among these approaches, the use of a metasurface is being increasingly suggested. Single negative metamaterials^26–33^ are often used to efficiently minimize mutual coupling by using unique permittivity and permeability features. Nevertheless, these metasurfaces, often positioned above arrays of antennas, have the capability to increase the size of the array profiles. Some of them were also used for wideband decoupling.

This study suggests the use of a two-element MIMO antenna with a metasurface absorber to improve isolation in mm-wave 5G communication devices, taking into account the constraints seen in the preceding antenna designs. To increase the channel capacity capability, a single-element antenna is transformed into an array structure of a 1 × 2 element. In order to optimize signal amplification and minimize interference between the MIMO components, a metasurface consisting of regularly arranged 1 × 3 Circular Split Ring (CSR) shaped cells is positioned above the MIMO antennas. The suggested antenna is designed to operate in the mm-wave frequency spectrum, specifically covering the range of 32.50–42.5 GHz. Furthermore, the insertion of the metasurface absorber leads to an enhancement in isolation.

## The design process and antenna geometry

### Antenna design geometry

**Fig. 1:**
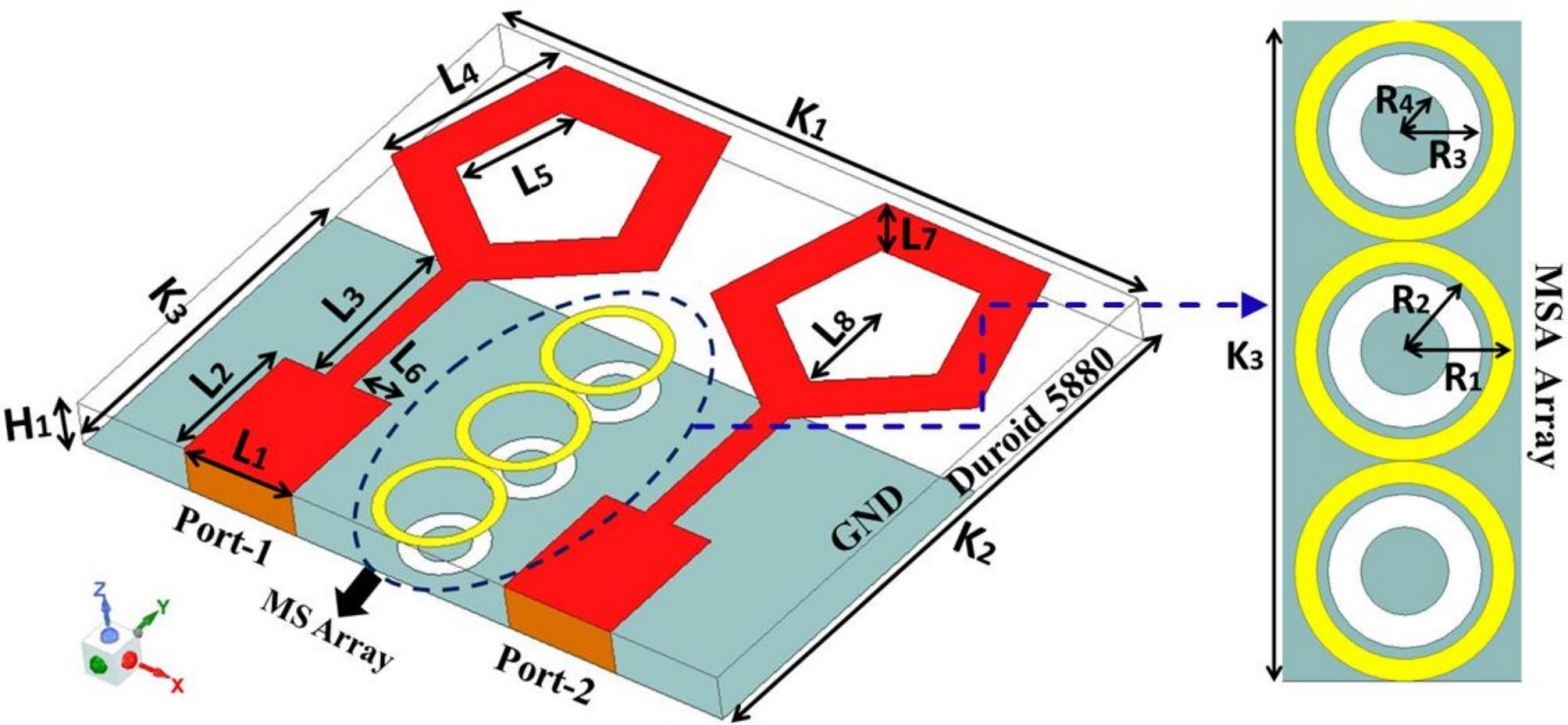
MIMO antenna with metasurface absorber array.

**Fig. 2:**
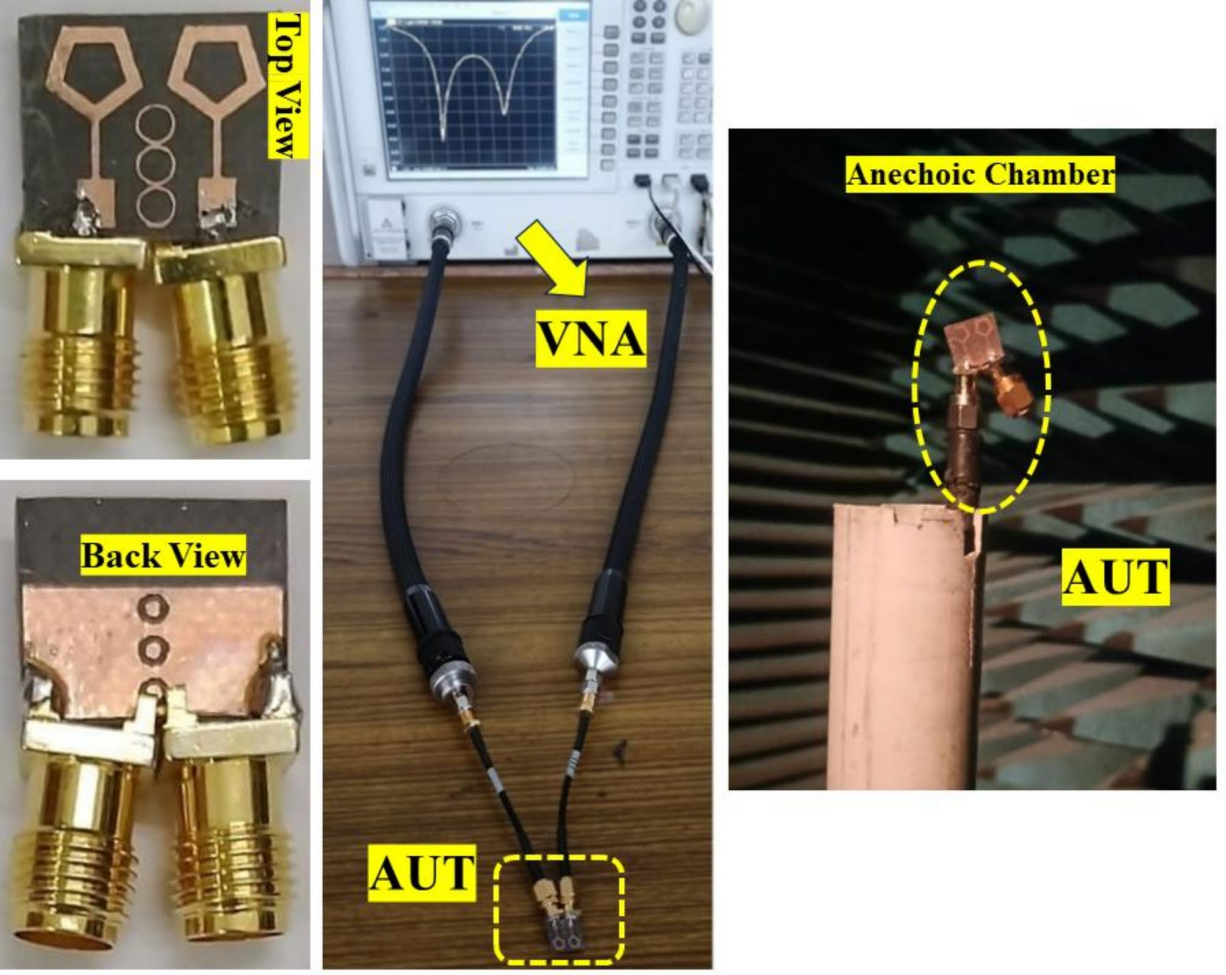
Images of the suggested design, both fabricated and taken with experimental setup.

Figures 1 and 2 depict the proposed MIMO antenna’s geometrical arrangement as well as photos that were produced using the antenna fabrication. The front diagonal view of a MIMO antenna module with metasurface absorber (MSA) array elements is shown in Fig. 1. Figure 2 displays photos of the antennas that were manufactured together with the specifications of the AUT. With a total size of 12 × 10 × 0.8 mm^3^, the complete compact antenna module with MSA elements has been presented and is printed using Rogers RT duroid 5880 high-frequency laminates. These laminates have a thickness of 0.8 mm and a relative permittivity of 2.2 to ensure optimal performance. The final proposed module consists of 2 identical pentagonal ring shape patches fed with a stepped impedance line and a double ring loaded 1 × 3 metasurface absorber array. The proposed antenna’s geometrical characteristics and dimensional specifications are described in Table 1.

**Table 1:** The dimensional specification of different geometrical parameters.

Parameters	Dimensions (in mm)	Parameters	Dimensions (in mm)
L_1_	2	L_8_	1.5
L_2_	2.4	K_1_	12
L_3_	2.78	K_2_	10
L_4_	2.78	K_3_	6
L_5_	0.87	H_1_	0.8
L_6_	0.75	R_1_, R_2_	1, 0.8
L_7_	0.87	R_3_, R_4_	0.7, 0.4

### Single antenna design

**Fig. 3:**
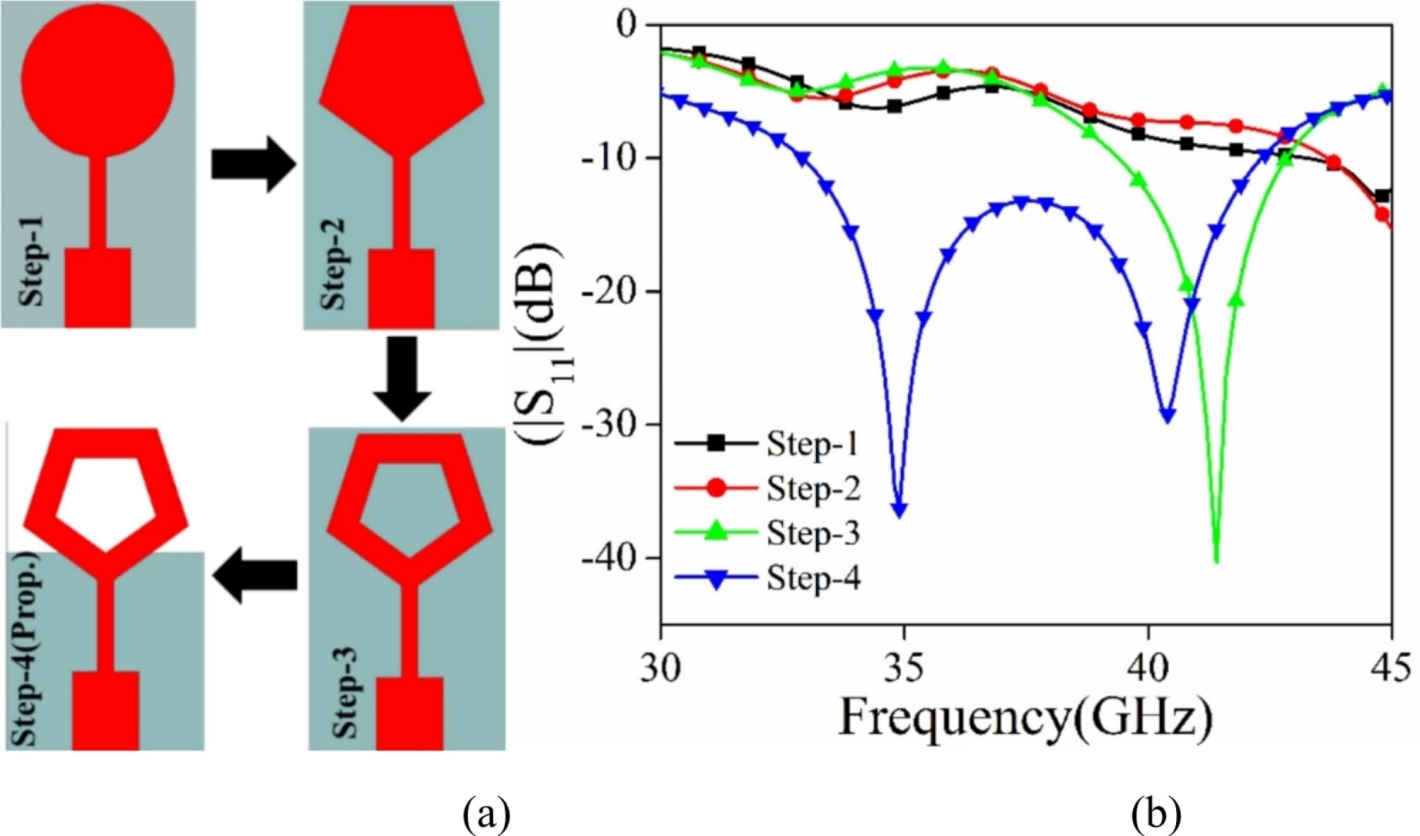
(**a**) The progression stages for the suggested antenna component. (**b**) |S_11_| (dB) plot for different stages.

This section examines and explores many phases of development of the proposed single antenna element. The final design of the suggested antenna is achieved by four distinct processes (Step 1 to Step 4). A comparison analysis is conducted focusing on the |S_11_| (dB) parameter, as shown in Fig. 3. Upon examining picture 1, it is evident that the formation of a pentagonal ring structure leads to resonance at higher frequencies in step 3. To further improve the bandwidth, the notion of a defective ground structure is added in step 4. Due to the establishment of the DGS, a secondary resonance is detected at a lower frequency. The total bandwidth of the suggested pentagonal shape structure with stepped impedance feeding and defected ground structure (DGS) is 32.5–42.5 GHz, with a span of 10 GHz.

## Design of metabsorber and mutual coupling suppression

### Designing of metasurface absorber

**Fig. 4:**
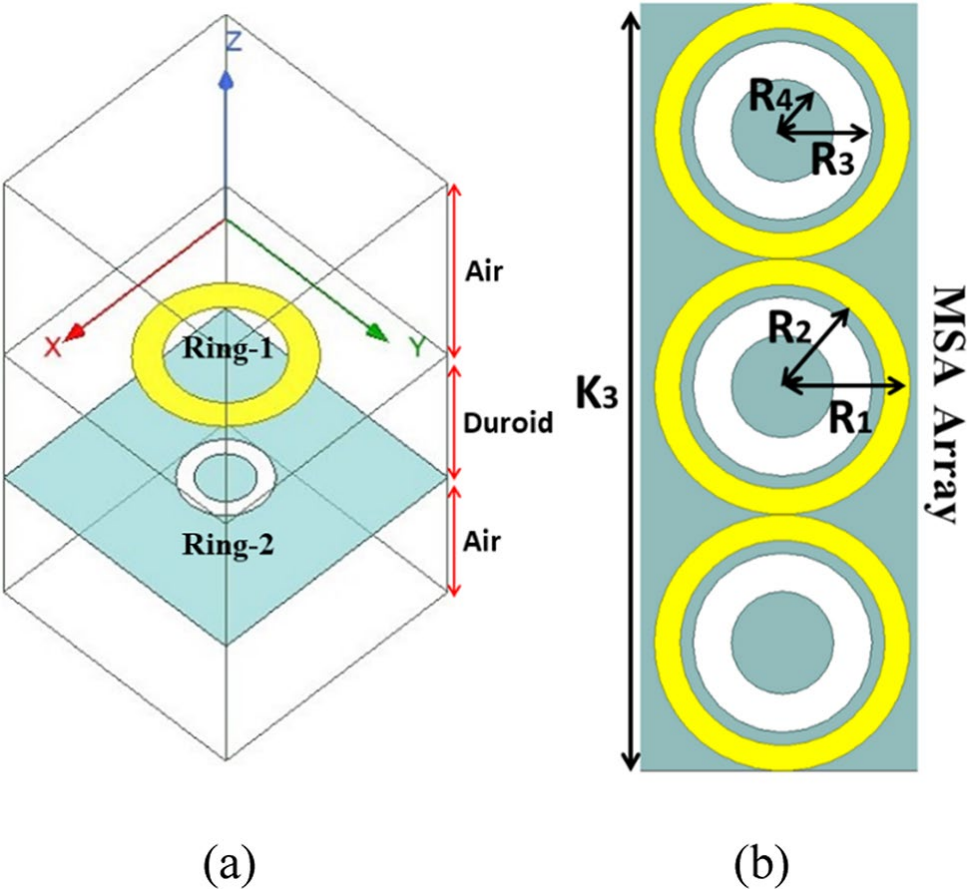
(**a**) Configuration of the suggested MA unit cell in the simulation, (**b**) Diagram illustrating the structure of the metamaterial absorber unit cell being suggested.

The electromagnetic absorber using metasurfaces is developed on a duroid substrate with a thickness of 0.8 mm as shown in Fig. 4. The substrate has a relative permittivity of 2.2 and a loss tangent of 0.0009. The design comprises two circular rings. The first ring, known as the conducting ring 1, is printed on top of the substrate. The second ring, referred to as ring 2, is created by etching a circular groove in the ground surface. The proposed absorber is evaluated using ANSYS HFSS, a software that models the unit cell with periodic boundary conditions. This enables the examination of an unlimited range of absorbers. The formula to determine absorption is A = 1 − |S_11_|^2^ − |S_12_|^2^, where A represents absorption and |S_11_|^2^ and |S_12_|^2^ represent the reflected and transmitted power respectively^34^.

**Fig. 5:**
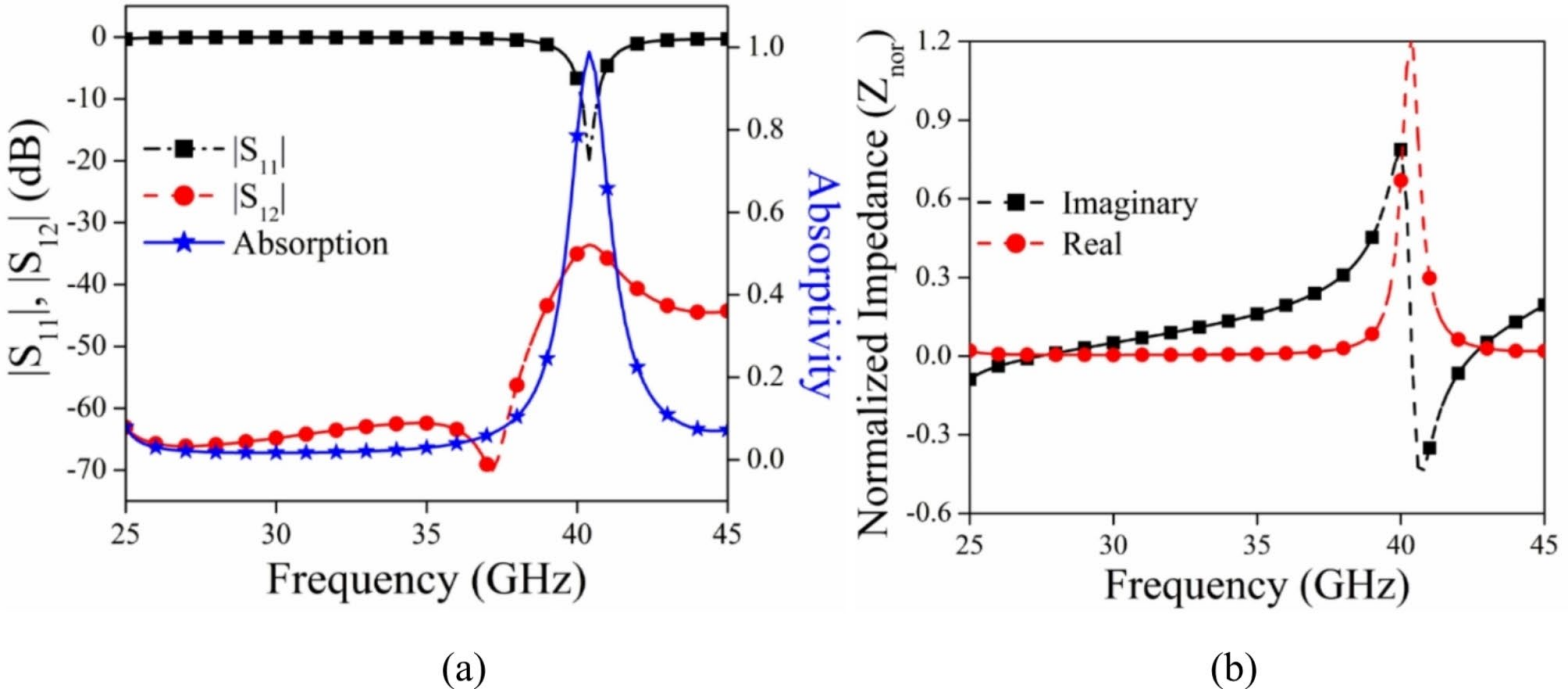
(**a**) Graph depicting the absorptivity, reflectivity, and transmissivity of the MSA when the incident angle is normal. (**b**) Graph illustrating the normalized impedance.

Figure 4 displays the geometric arrangement and dimensional details of the MSA, with R_1_ = 1 mm, R_2_ = 0.8 mm, R_3_ = 0.7 mm, and R_4_ = 0.4 mm. The metasurface’s absorptivity is determined by its reflectivity and transmissivity qualities. In the suggested design, the majority of the ground component acts as a conductor, resulting in a transmissivity |S_12_|^2^ ≈ 0. The graph in Fig. 5a displays the absorptivity, reflectivity, and transmissivity of the suggested MA when it is exposed to normal incidence. Upon examining Fig. 5a, it is evident that the absorption peak is over 100% at 40 GHz. Additionally, the magnitudes of |S_11_| are quite low, whereas |S_12_| is close to zero. To provide further evidence for the absorptivity characteristics of the suggested metasurface, we have computed the normalized impedance Z_nor_ = $\sqrt{{\varepsilon }_{r}/{\mu }_{r}}$ and included the corresponding graph in Fig. 5b. Upon analyzing Fig. 5b, it is evident that the real part of the impedance closely approximates 1, which is in close proximity to the normalized characteristic impedance of free space (normalized to 377 Ω). Additionally, the imaginary part is nearly zero, indicating the metasurface’s perfect absorption attribute. The deep learning approach is used to study the change in the absorption peak of MSA for varied dimensional values of R_1_, R_2_, R_3_, and R_4_.

## Deep learning analysis to attain the maximum absorption by optimizing the MSA

Deep Learning is utilized to optimize the dimensional configuration of the metasurface to achieve the maximum absorption of electromagnetic waves in the band of interest. To achieve this goal, Deep Neural Network (DNN), Long Short-Term Memory (LSTM), and Transformer models are utilized for the training and testing of models with the datasets generated by HFSS. Three different datasets are generated by performing parametric analysis in HFSS. The first dataset is generated for the reflection coefficient (|S_11_|) while the second one is generated for (|S_12_|). Similarly, the third dataset is generated for absorption (A).

### Dataset generation

All three datasets are generated by varying the radius of two circular rings on two different layers of metasurface. As shown in Fig. 4b, the Ring-1 has two radii R_1_ and R_2_. The radius R_1_ is varied from 0.5 mm to 1 mm with a linear step of 0.1 mm while the R_2_ is varied from 0.4 mm to 0.9 mm with the same linear step of 0.1 mm. The Ring-2 also has two radii R_3_ and R_4_. It is shown in Fig. 4b. The radius R_3_ and R_4_ are also varied from 0.2 mm to 0.7 mm and 0.1 mm to 0.6 mm respectively with a linear step of 0.1 mm. The variation of R_1_, R_2_, R_3_, and R_4_ produces the 1296 combinations. The frequency sweep for the dataset generation is taken from 32.5 GHz to 42.5 GHz with a linear step of 0.1 GHz; therefore producing 101 points. With all these varying parameters, a total of 1,30,896 dataset points have been generated for S_11_ (dataset-1), S_12_ (dataset-2), and Absorption (dataset-3). All three datasets are divided into two parts i.e. training dataset comprising 80% (104,717) dataset points and testing dataset comprising 20% (26,179) dataset points.

### Model architecture and evaluation

Two loss function parameters MSE (Mean-Squared Error), and MAE (Mean Absolute Error) are used in performance analysis. The deep Neural Network model is designed with three hidden layers, one input and one output layer. The activation function is a Rectified linear activation unit (ReLU). The initial learning rate of the model is 0.001 and the total epochs are 100. For the training purposes of the DNN model, Adam optimizer is utilized.

Furthermore, a sequential LSTM model is designed with two LSTM layers (100 and 50 units), dropout layers (rate 0.2), a dense layer (32 units, ReLU), and an output layer. The activation functions for LSTM layers are tanh and sigmoid internally, and Dense layer uses ReLU, while the output layer uses linear. Adam is used as an optimizer Total 100 epochs, batch size 32, is used for training.

Thereafter, a simplified Transformer model is designed with several Transformer encoder blocks (head size 256, 4 heads, feed-forward dimension 4, dropout rate 0.2), a Global Average Pooling layer, dense layers (one with 128 units, ReLU, and a dropout rate of 0.3), and an output layer. The activation functions for the dense layers use ReLU, while the output layer uses a linear activation function. Adam is used as the optimizer. A total of 100 epochs, with a batch size of 32, is used for training.

### Optimization of R_1_, R_2_, R_3_ and R_4_

For each combination of R_1_, R_2_, R_3_ and R_4_ in the given frequency range of 32.5 to 42.5, Limited-memory Broyden-Fletcher-Goldfarb-Shanno with Box constraints (L-BFGS-B) method is applied for optimization in the generated dataset to obtain the maximum absorption. To find the optimal value of R_1_, R_2_, R_3_, and R_4_; all three models: the Deep Neural Network (DNN), LSTM model, and Transformer model, targeting the minimization of the prediction error across a frequency range from 32.5 to 42.5 GHz is applied. The optimization process uses the scipy.optimize.minimize function with the L-BFGS-B method. For each model, an objective function is defined to calculate the average error between the predicted values (S11, S12, and Absorption) and the desired values across all frequencies. The parameters R_1_, R_2_, R_3_, R_4_ are adjusted to minimize this error. Initial guesses for the parameters are set to [0.5, 0.4, 0.2, 0.1], and the bounds for the parameters are [(0.5, 1.5), (0.4, 1.4), (0.2, 1.2), (0.1, 1.1)]. The objective function involves creating feature sets for each frequency, scaling them, predicting the output using the respective model, and computing the squared error between the predictions and the desired values. The optimization results in the optimized parameters for each model, which are 1.0 mm for R_1_, 0.9 mm for R_2_, 0.7 mm for R_3_ and 0.6 mm for R_4_.

### Model comparison and results

**Fig. 6:**
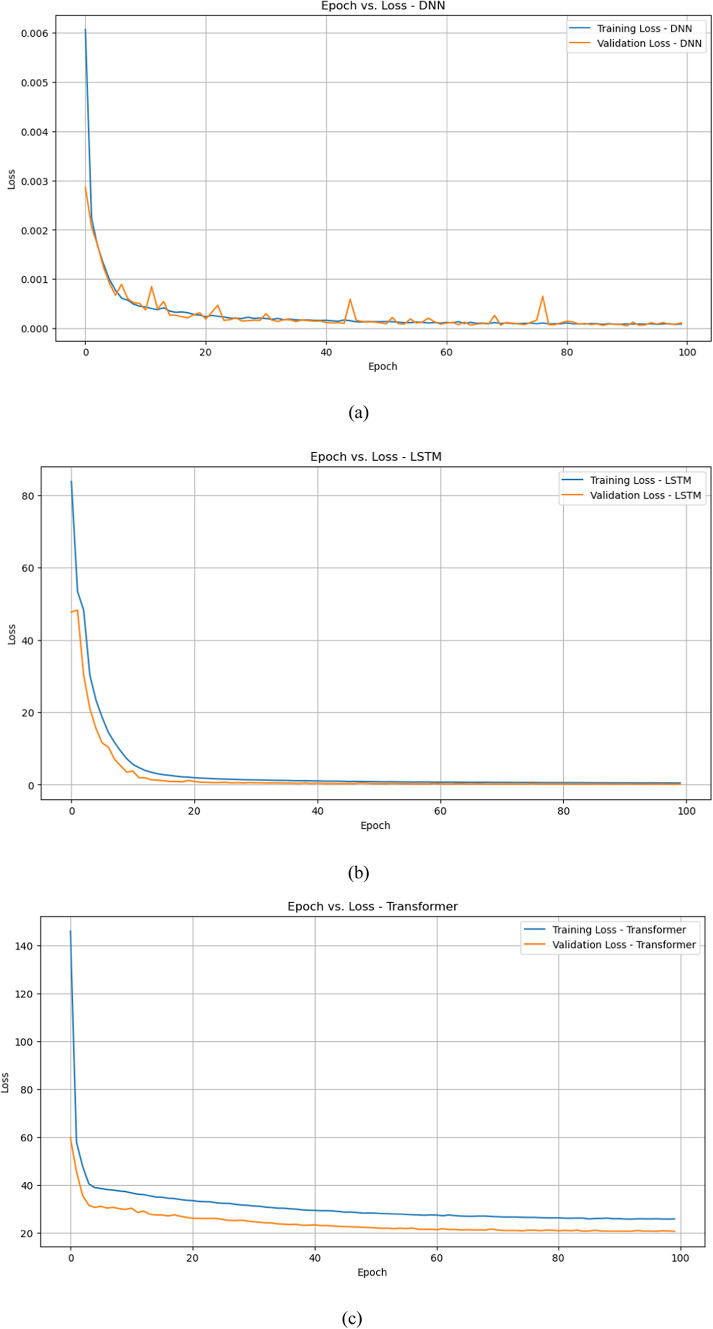
Epoch versus training and validation loss (**a**) DNN model (**b**) LSTM model (**c**) Transformers model.

Figure 6a–c shows the training loss and validation loss for DNN model, LSTM model, and Transformer model respectively. DNN model exhibits the lowest training and validation losses, indicating it learns the training data efficiently and generalizes well to the validation data. The LSTM model shows a substantial reduction in training and validation losses, with the validation loss slightly lower than the training loss, indicating effective learning and generalization with minimal overfitting. In the Transformer model, although the training and validation losses decrease, they remain higher compared to the DNN and LSTM models.

In conclusion, the DNN model performs the best in terms of both training and validation loss, followed by the LSTM model, which also demonstrates strong learning and generalization capabilities. The Transformer model, while showing a significant reduction in loss, lags behind in performance compared to the other two models.

**Fig. 7:**
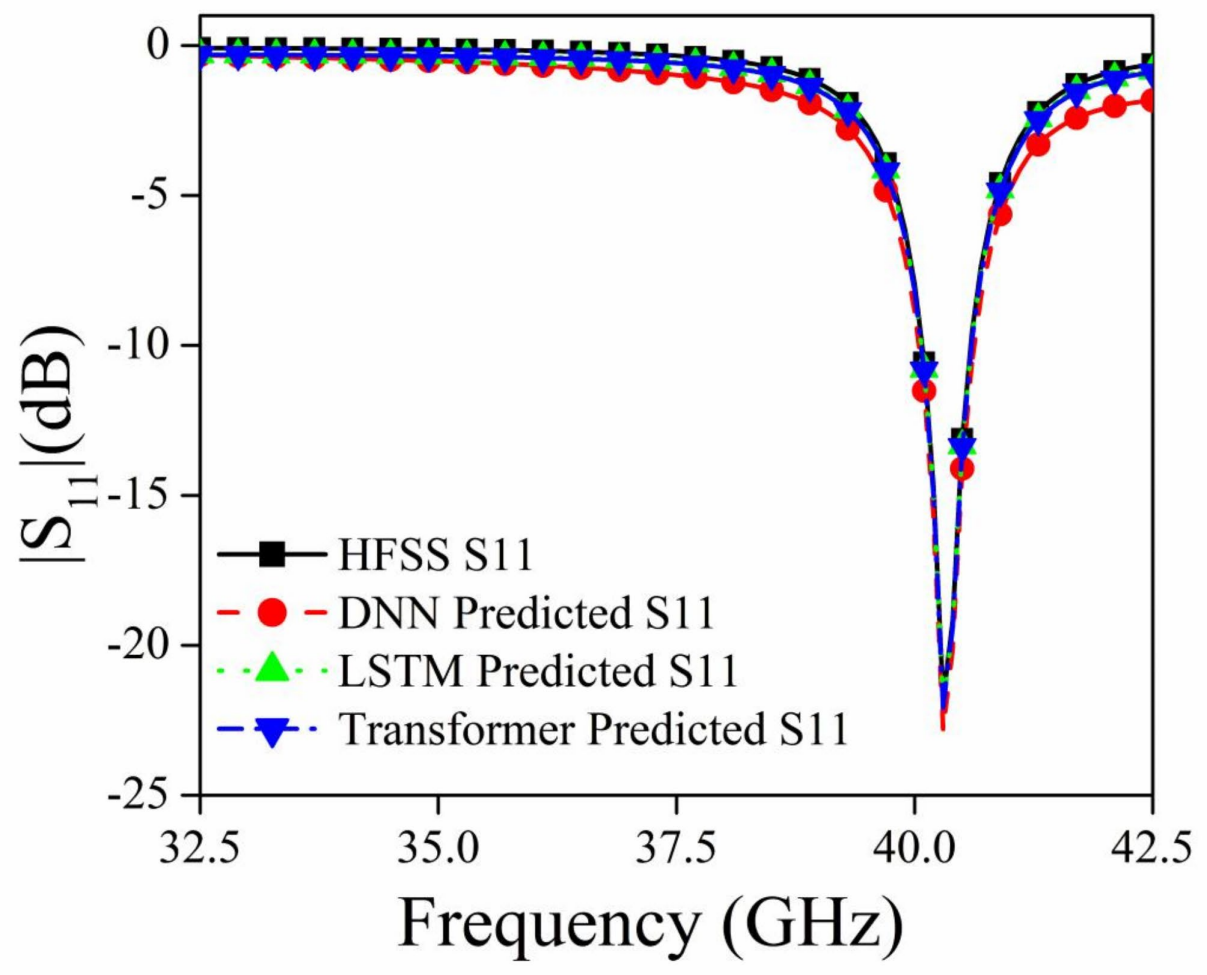
Comparison of different deep learning models and HFSS results for |S_11_|.

**Fig. 8:**
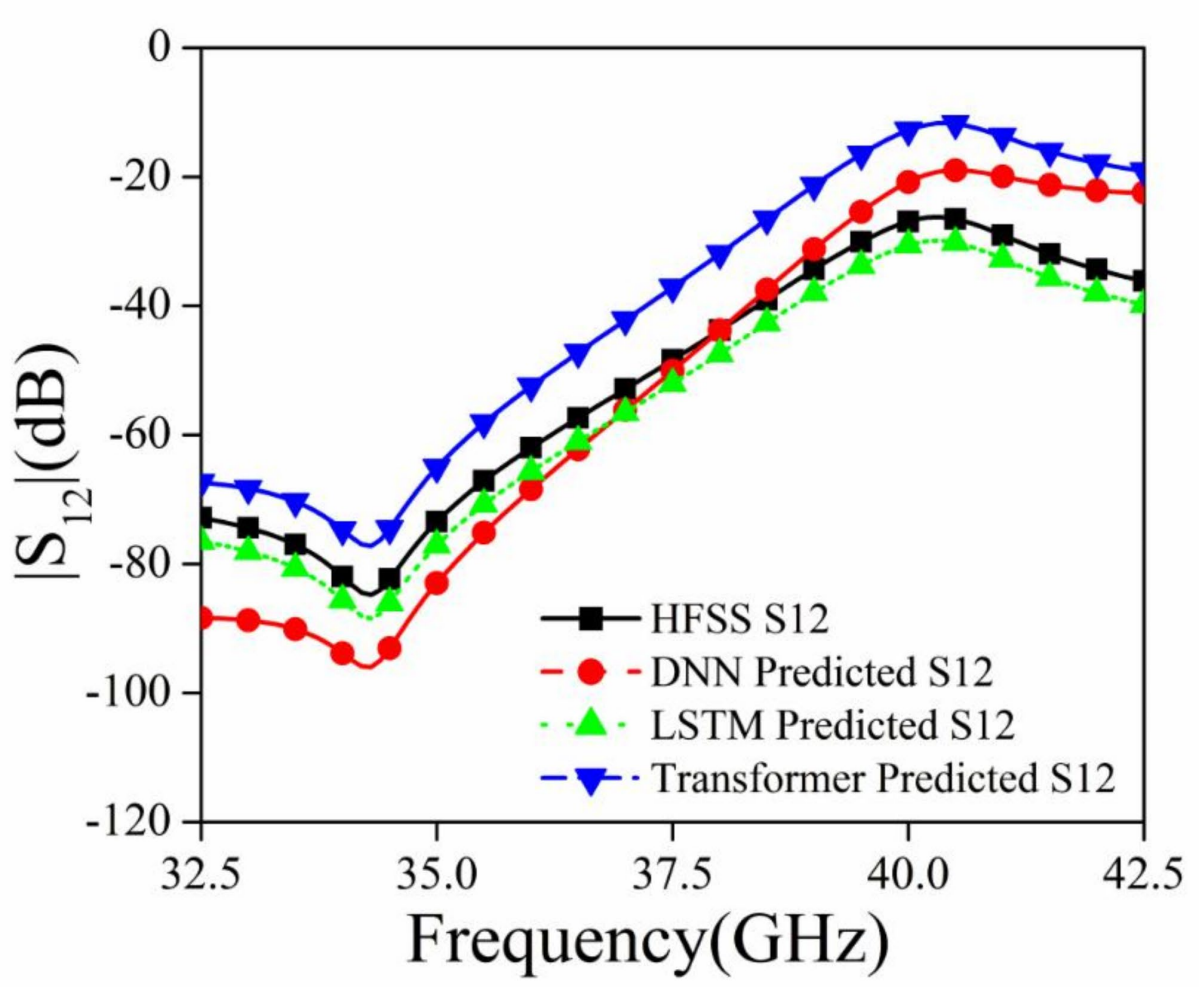
Comparison of different deep learning models and HFSS results for |S_12_|.

**Fig. 9:**
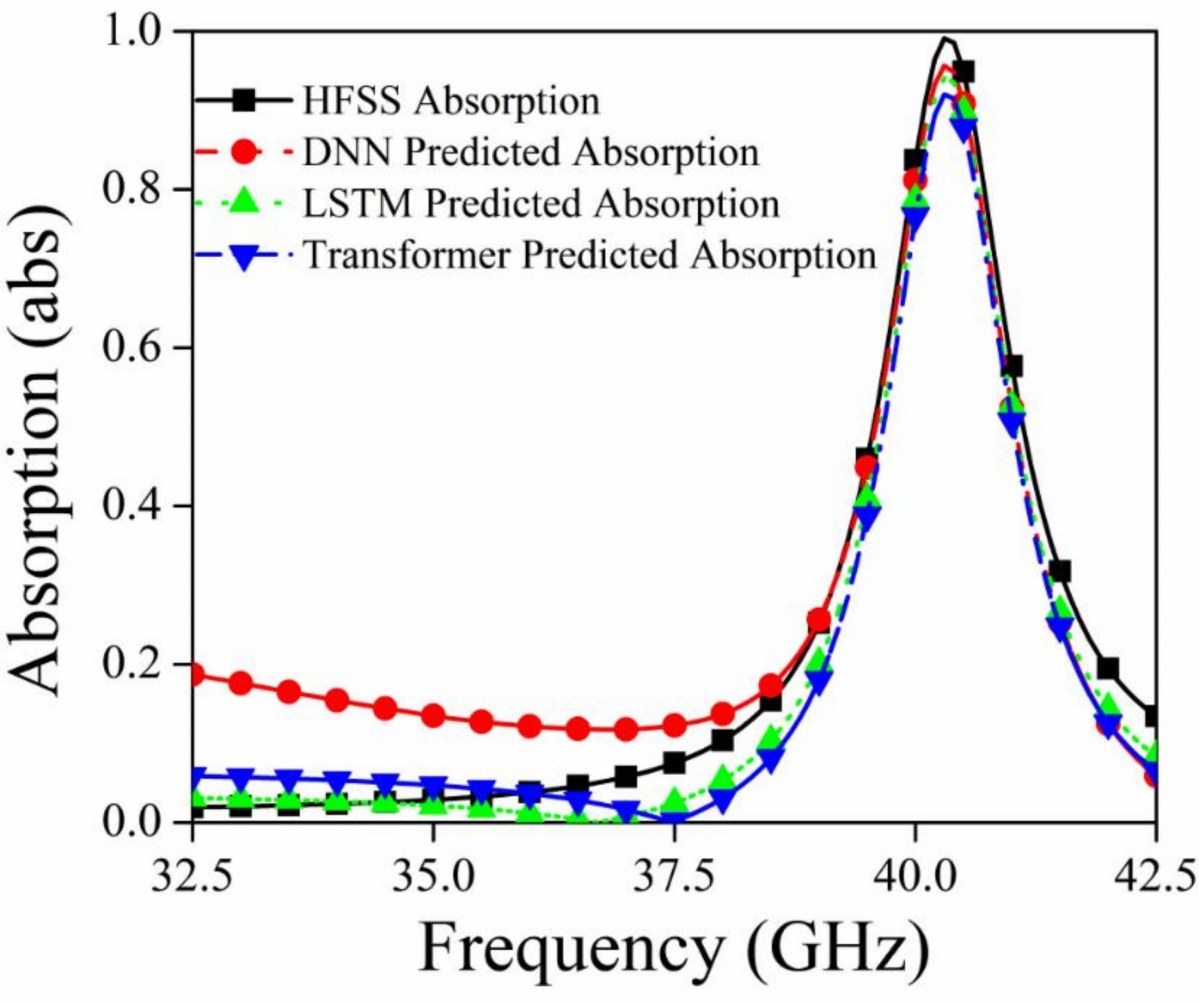
Comparison of different deep learning models and HFSS results for absorption.

Figures 7, 8, and 9 shows the S_11_ versus Frequency, S_12_ versus Frequency, and Absorption versus Frequency comparison charts respectively, at optimal values of R_1_ = 1.0 mm, R_2_ = 0.9 mm, R_3_ = 0.7, and R_4_ = 0.6 mm for all three models and HFSS results.

The DNN model demonstrates the best performance across all three datasets (Absorption, S_11_, and S_12_), closely matching the HFSS simulation results. It accurately captures the peaks, troughs, and overall trends, indicating its robustness and suitability for this prediction task. The LSTM model shows relatively compromising performance in capturing the significant variations in the HFSS results. The Transformer model also shows limitations in capturing the detailed variations in the HFSS results. In conclusion, the DNN model outperforms both the LSTM and Transformer models in accurately predicting the desired parameters (Absorption, S_11_, and S_12_) when compared to the HFSS simulation results.

The computational complexity of the Deep Neural Network (DNN) model used in this study can be described as follows:

The computational complexity of a fully connected DNN is determined primarily by the number of neurons in each layer and the number of connections between layers. For a DNN with L layers, where each layer $i$ has ${n}_{i}$ neurons, the number of computations (multiplications and additions) for a forward pass is approximately:

$$O\left(\sum _{i=1}^{L-1}{n}_{i}\cdot {n}_{i+1}\right)$$

In our case, the DNN consists of three hidden layers with a fixed number of neurons and an output layer for predicting |S_11_|, |S_12_|, and Absorption. This architecture results in a low computational cost. The simplicity of matrix multiplications makes the DNN computationally efficient for our task.

The comparison between the DNN model and other simpler machine learning models such as Decision Tree, Random Forest, and XGBoost, followed by a justification for using the DNN model is shown in Table 2.

**Table 2:** Model comparison.

Model	Ability to handle non-linearity	Accuracy (based on task)	Generalization
Decision tree	Moderate (can split non-linearly at nodes)	Moderate accuracy for complex tasks	Prone to overfitting on deep trees
Random forest	MODERATE TO HIGH	Higher than decision tree	Better generalization via averaging
XGBoost	High	Generally high accuracy	Good generalization through boosting
DNN	Very high	Very high (matches HFSS well)	Excellent with proper tuning and regularization

Key justification points for using DNN over simpler models:

1. Handling complex non-linear relationships: Decision trees and random forests handle non-linearity to a moderate extent, but they rely on simple feature splits, which may not be able to model complex interactions between variables as effectively as a DNN. The DNN can model non-linearity more accurately because of its deep architecture and ReLU activations.
2. Accuracy in this task: As demonstrated in the results, the DNN performs very closely to the HFSS (simulation) results in terms of accuracy. While Random Forest and XGBoost are generally strong in terms of accuracy, they do not perform as well for this specific task because they lack the depth and architecture necessary to model highly complex relationships.
3. Generalization: Simpler models like Decision Trees can easily overfit if the trees are too deep, while Random Forests mitigate this issue by averaging predictions across multiple trees. However, DNN offers similar generalization capabilities when regularized properly (e.g., using dropout), making it a better choice for this task without the drawbacks of overfitting.

In conclusion, while simpler models like Decision Trees, Random Forests, and XGBoost can be effective for a range of tasks, the DNN model offers superior accuracy, generalization, and the ability to model complex non-linear relationships, which is crucial for the accurate prediction of |S_11_|, |S_12_|, and Absorption in this study. This justifies the inclusion of the DNN over these simpler alternatives.

This detailed analysis of all three deep learning models suggests that the optimization of R_1_, R_2_, R_3_, and R_4_ has produced the most accurate values for the radius of both Ring-1 and Ring-2 which can be utilized for further analysis of the metasurface.

### Mutual coupling suppression

**Fig. 10:**
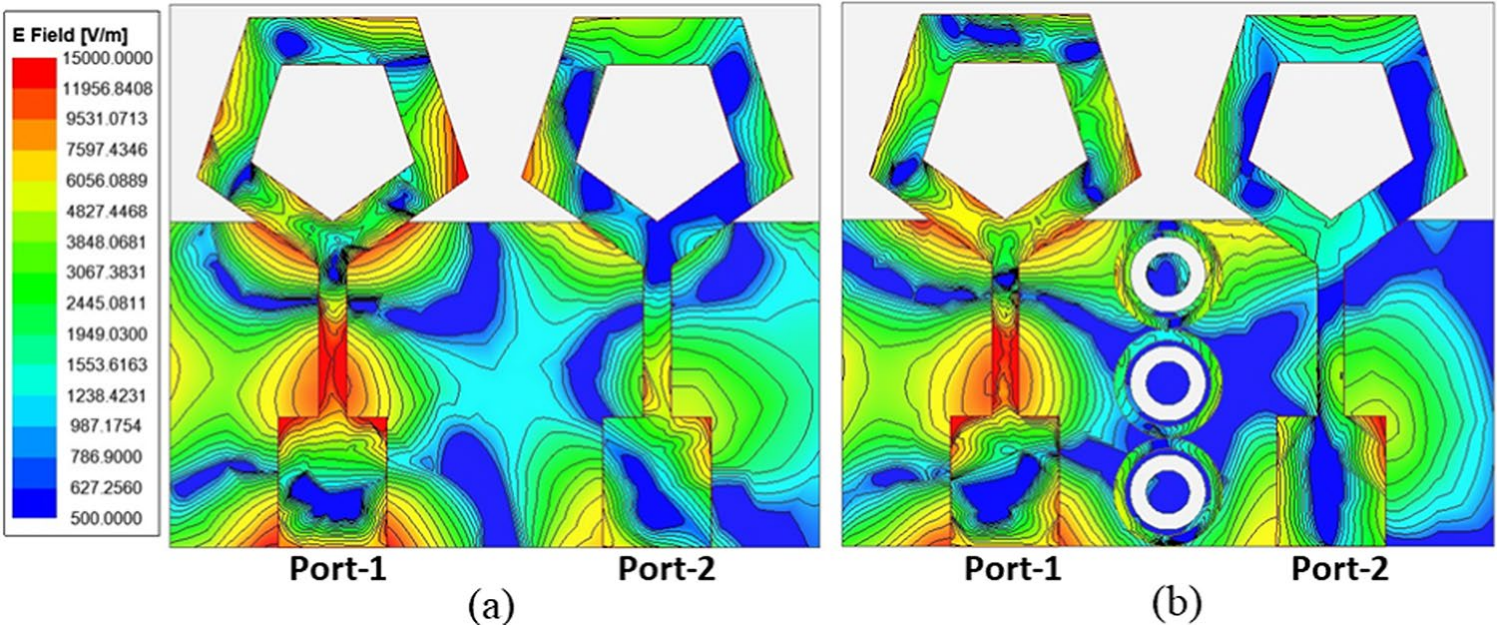
Electric filed strength plot representing the mutual coupling between the MIMO antenna (**a**) without MSA, (**b**) with MSA.

**Fig. 11:**
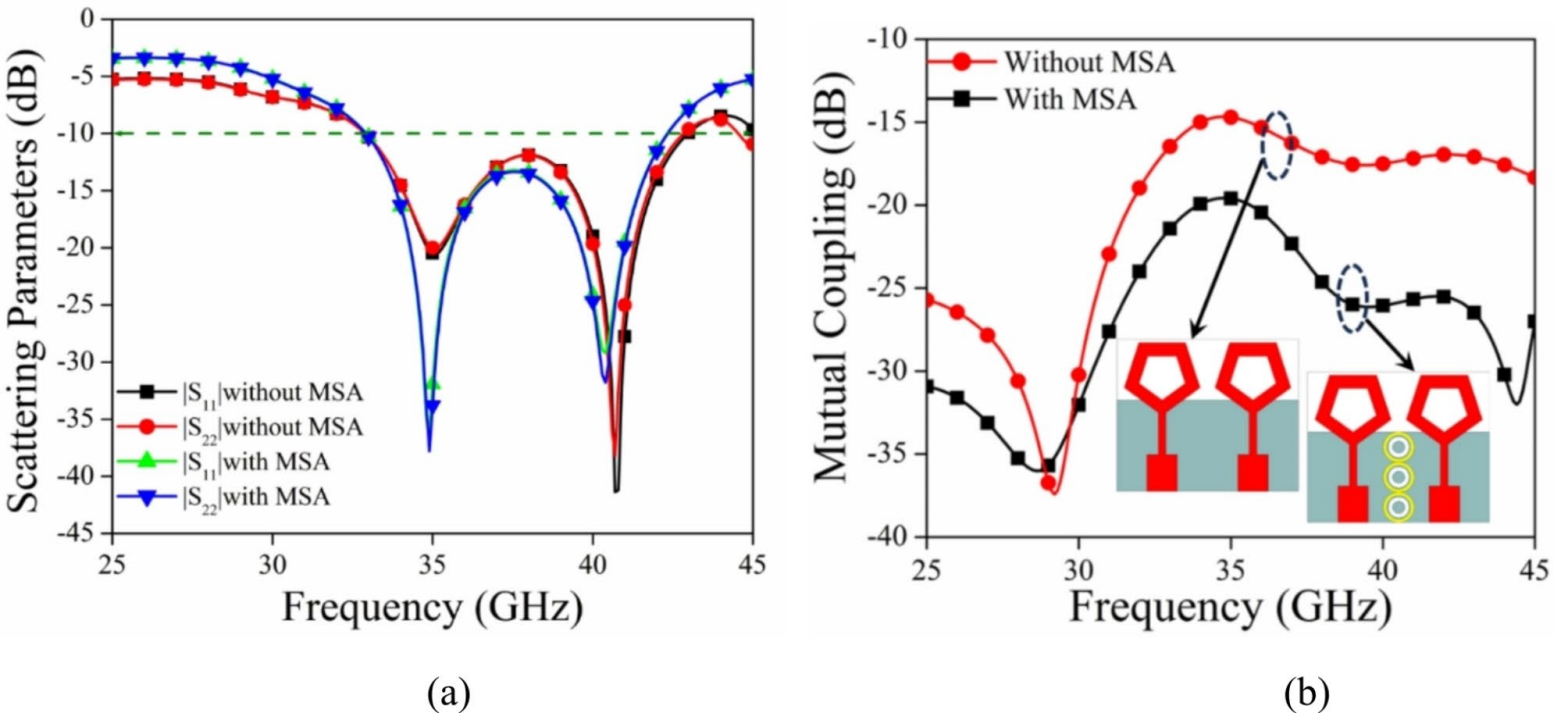
(**a**) Scattering parameters and (**b**) mutual coupling of the MIMO antenna with and without MSA.

In order to enhance the isolation between the antenna elements, a 1 × 3 array of MSA is strategically positioned between them, as shown in Fig. 1. The plot of electric field strength and the values of scattering parameters for the MIMO antenna are displayed in Figs. 10 and 11 respectively, both with and without the meta-absorber. Figure 10a illustrates the noticeable coupling between ports 1 and 2 when the meta-absorber is not present. However, the presence of the meta-absorber reduces this coupling by absorbing as much electromagnetic energy as it can, preventing it from traveling between port 1 and port 2 (cf. Fig. 10b). Based on the graph of Fig. 11a, it can be seen that the impedance bandwidth values for both configurations are very similar, with slight variations in resonance frequency when the metasurface is introduced near the antenna elements. This is due to the capacitive coupling between the radiation and metasurface, resulting in a change in resonance frequency. While the mutual coupling (|S_12_|) between antenna components shows a notable improvement in isolation, going from − 15 dB to − 26 dB, it is evident that this is due to the effective absorption of the metasurface absorber (cf. Fig. 11b).

## Results and discussion

This section specifically addresses three crucial points: (i) Conducting a comparison between simulated and observed results. (ii) Quantifying diversity performance using calculations. (iii) Evaluating the performance of the proposed module in comparison to other metasurface-inspired antenna modules documented in existing literature. Figure 2 shows the antenna prototype undergoing testing with the vector network analyzer (VNA) to verify its simulated performance.

### Scattering characteristics

**Fig. 12:**
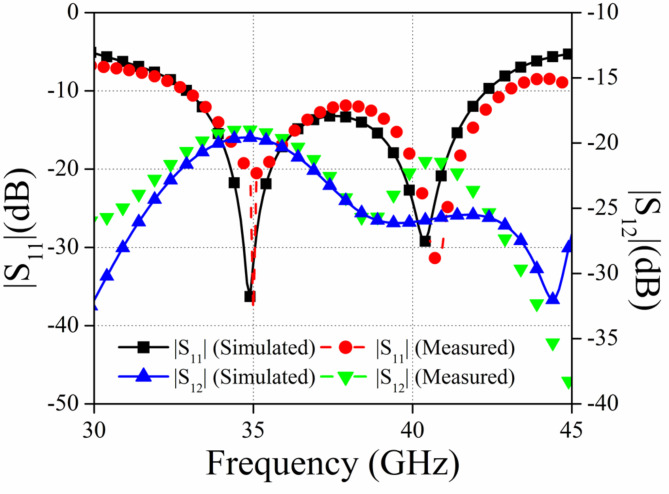
The simulated and measured scattering parameters for the proposed configuration.

Figure 12 illustrates the measured and simulated fluctuations of the suggested MIMO antenna’s |S_11_| and |S_12_|. Upon examining Fig. 12, it is evident that the proposed antenna module design exhibits an impedance bandwidth of 32.5 GHz to 42.5 GHz (simulated) and 32.9 GHz to 43.0 GHz (measured). Upon examining Fig. 12, it is evident that the results of both the simulated and observed scattering parameters are consistent. The minor disparities between the simulated and measured results may be attributed to the tolerance in manufacture and the inadequate soldering of the connections.

### Radiation pattern

**Fig. 13:**
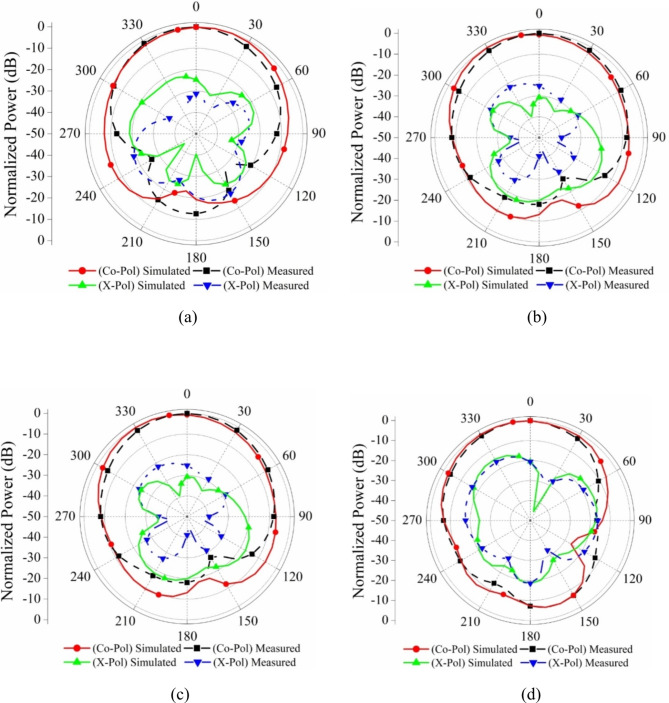
2-Dimensional radiation pattern port wise: Port 1 (**a**) at 35 GHz (**b**) at 40 GHz, Port 2: (**c**) at 35 GHz (**d**) at 40 GHz.

Figure 13 shows the normalized far-field radiation pattern of the proposed antenna resonating at 35 GHz and 40 GHz respectively, in the frequency band of 32.5–42.5 GHz at two different ports. From the observations of the plot, it is noticed that the measurement was done with one port active at one time keeping the other port with matched terminated by 50 Ω impedance (It is because of the non-availability of multi-port measurement in the anechoic chamber at the testing facility lab). A strong correlation has been seen between the simulated and measured far-field radiation patterns. At some angles, the cross-polarization is higher than at other angles of radiation. However, in the majority of the radiating area, it has a significantly smaller value compared to the level of co-polarization. All of these patterns are monitored inside the anechoic chamber, where additional ports are terminated with a matching load.

### Gain and radiation efficiency

**Fig. 14:**
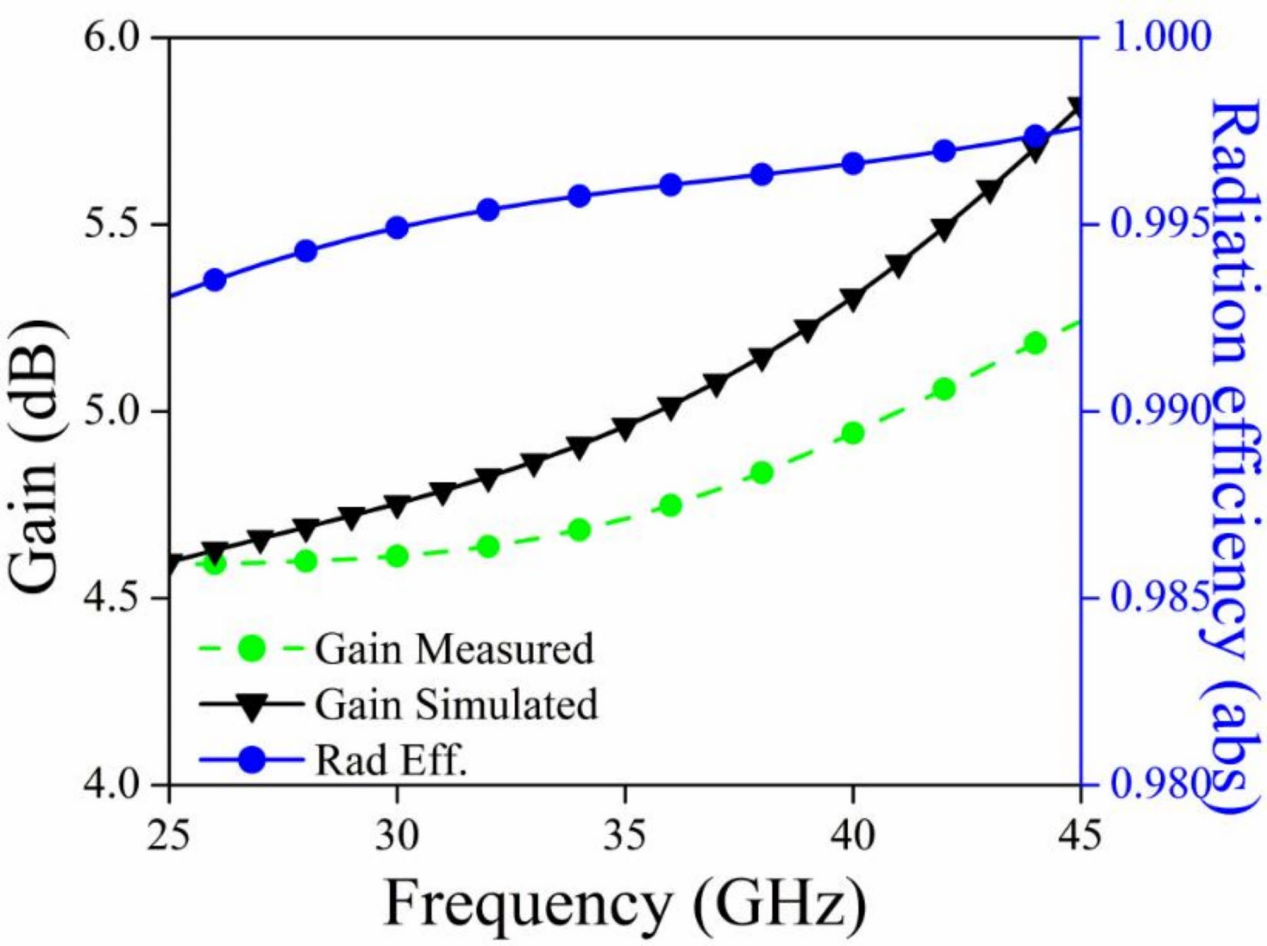
Gain (Sim/Mes) and radiation efficiency plot.

Figure 14 displays the measured and simulated gain of the MIMO antenna, as well as the radiation efficiency plot. The operational frequency range achieves a net improvement of 4.5–5.5 dB, with an optimal gain of 5.5 dB at 42.5 GHz. Two conclusions can be drawn from analyzing this plot: (a) an increase in frequency leads to an increase in gain because the effective aperture of an antenna is larger compared to the wavelength, and (b) the radiation efficiency exceeds 98% across the entire impedance bandwidth.

A method known as the Two Antenna Method is used in order to determine the gain of the proposed antenna. The following is a sequence of the measurements for antenna gain:

I. The planned antenna is installed in the broadside direction from the outside.
II. An antenna with a standard gain on the other side (whose gain is already known to us, often known as GT).
III. Once you have determined S_21_ at each frequency point, you will have obtained (PR-PT).
IV. The path losses are being determined by a factor of 20 log10 (4πR/λ).
V. The distance between the transmitting antenna and the receiving antenna is denoted by R.
VI. Determine the amount of cable loss by connecting the sending and receiving cables in a direct connection.
VII. Now, finally used Friss Equation formula given as follow:
  $${\text{G}}_{{\text{R}}} = {\text{S}}_{{21}} \left( {{\text{in dB}}} \right) + 20\log 10\left(-4\uppi {\text{R}}/\uplambda \right) - {\text{G}}_{{\text{T}}} + {\text{cable loss}}.$$

### Diversity characteristics

The performance of the proposed MIMO module diversity with metasurface absorber is assessed using metrics such as ECC, DG, CCL, and MEG. The formula for these metrics may be found in Table 3.

**Table 3:** The Standard equations to quantify the diversity characteristics of the MIMO module.

Diversity parameters	Standard expression	Method to calculate diversity parameter
Envelope correlation coefficient (ECC)^35^	$ECC_{s} = \left| {\frac{{\left| {S_{11}^{ * } S_{12} + S_{21}^{ * } S_{22} } \right|}}{{\left| {\left( {1 - \left| {S_{11} } \right|^{2} - \left| {S_{21} } \right|^{2} } \right)\left( {1 - \left| {S_{22} } \right|^{2} - \left| {S_{12} } \right|^{2} } \right)} \right|^{1/2} }}} \right|^{2}$	Scattering parameters
Envelope correlation coefficient (ECC)^35^	$ECC_{F} = \frac{{\left| {\iint\limits_{4\pi } {\left[ {E_{i} \left( {\theta ,\phi } \right) * E_{j} \left( {\theta ,\phi } \right)} \right]d\Omega }} \right|^{2} }}{{\iint\limits_{4\pi } {\left| {E_{i} \left( {\theta ,\phi } \right)} \right|^{2} d\Omega \iint\limits_{4\pi } {\left| {E_{j} \left( {\theta ,\phi } \right)} \right|^{2} d\Omega }}}}$	3-D field
Diversity gain (DG)^36^	$DG=10\sqrt{1-{ECC}^{2}}$	Scattering parameters
Channel capacity loss (CCL)	$$\begin{array}{*{20}l} {C_{loss} = - \log_{2} \det ({\uppsi }^{R} )} \hfill \\ {{\uppsi }^{R} = \left( {\begin{array}{*{20}c} {\psi_{11} } & {\psi_{12} } \\ {\psi_{21} } & {\psi_{22} } \\ \end{array} } \right),\;\;\psi_{11} = 1 - \left( {\left| {S_{11} } \right|^{2} + \left| {S_{12} } \right|^{2} } \right)} \hfill \\ {\psi_{22} = 1 - \left( {\left| {S_{22} } \right|^{2} + \left| {S_{21} } \right|^{2} } \right),\;\;\psi_{12} = - \left( {S_{11}^{*} S_{12} + S_{21}^{*} S_{12} } \right)} \hfill \\ {\psi_{21} = - \left( {S_{22}^{*} S_{21} + S_{12}^{*} S_{21} } \right)} \hfill \\ \end{array}$$	Scattering parameters
Mean effective gain	$$\begin{array}{*{20}l} {MEG_{1} = 0.5\left[ {1 - \left| {S_{11} } \right|^{2} - \left| {S_{12} } \right|^{2} } \right]} \hfill \\ {MEG_{2} = 0.5\left[ {1 - \left| {S_{12} } \right|^{2} - \left| {S_{22} } \right|^{2} } \right]} \hfill \\ \end{array}$$	Scattering parameters

**Fig. 15:**
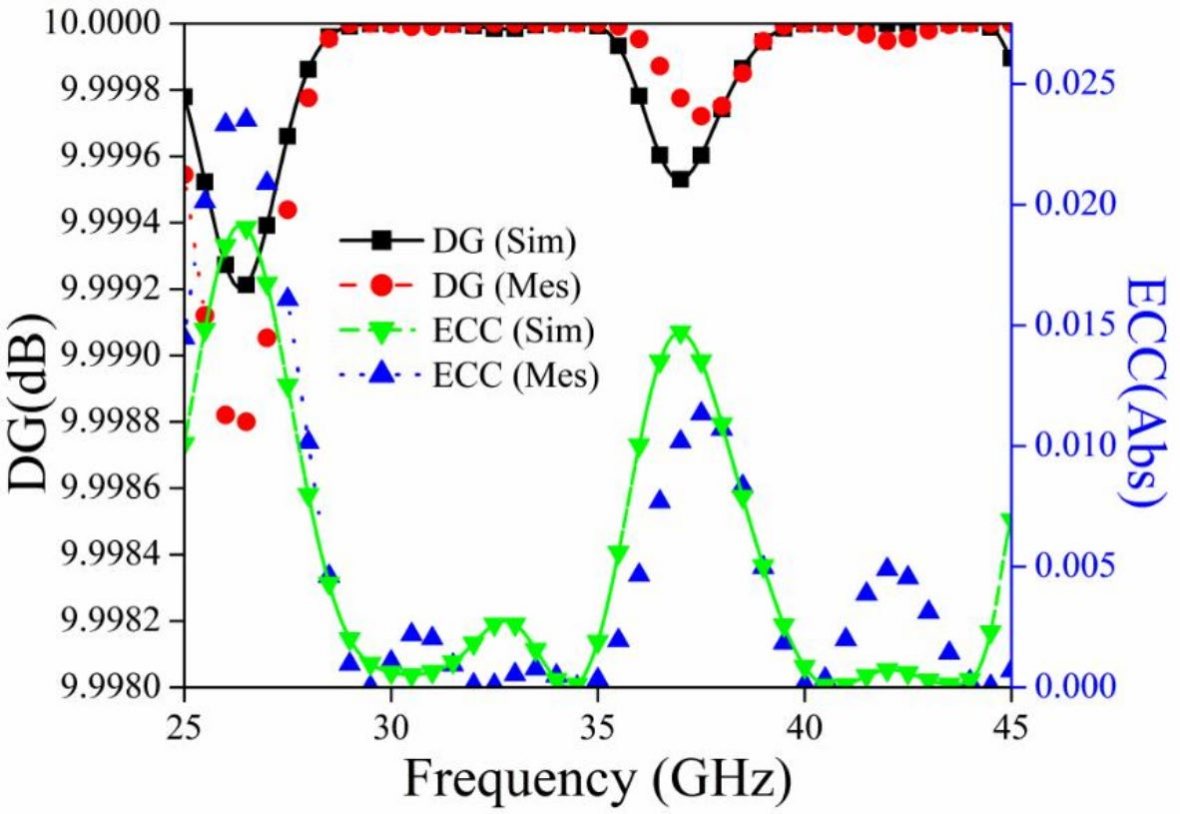
ECC and DG graph for the proposed MIMO antenna module.

As far as is known, the 3D radiation pattern at the target frequency provides correct data for measuring the diversity performance of an antenna. This is because MIMO antennas are utilized for multipath reflections. Radiated fields and s-parameters are therefore used to compute the ECC. For the MIMO antenna system, the ECC values computed by radiated fields are more practical than the s-parameters values because ECE includes all types of losses when field calculations are performed. It is also possible to determine the ECC values using the scattering parameters-based calculation if the radiation efficiency of the MIMO antenna is more than 90%. From the perusal of Fig. 9, it is clear that the radiation efficiency of the proposed MIMO antenna is more than 98% so the scattering parameters method can be utilized. The equation for ECC is mentioned in Table 3, which may be derived from MIMO architecture radiation pattern data and scattering parameters, and the pertinent graph is mentioned in Fig. 15. The value of ECC for the proposed MIMO antenna is less than 0.015 for the reported impedance bandwidth.

The second important parameter to check the independency of MIMO antenna elements is diversity gain (dB). The value of DG should be calculated with the help of the equation mentioned in Table 3 and the value of it should be high (10 dB) for the proposed impedance bandwidth of 32.5 GHz to 42.5 GHz). From the perusal of Fig. 15 it is clear that the value of DG is approximately 10 dB for the aforementioned bandwidth.

**Fig. 16:**
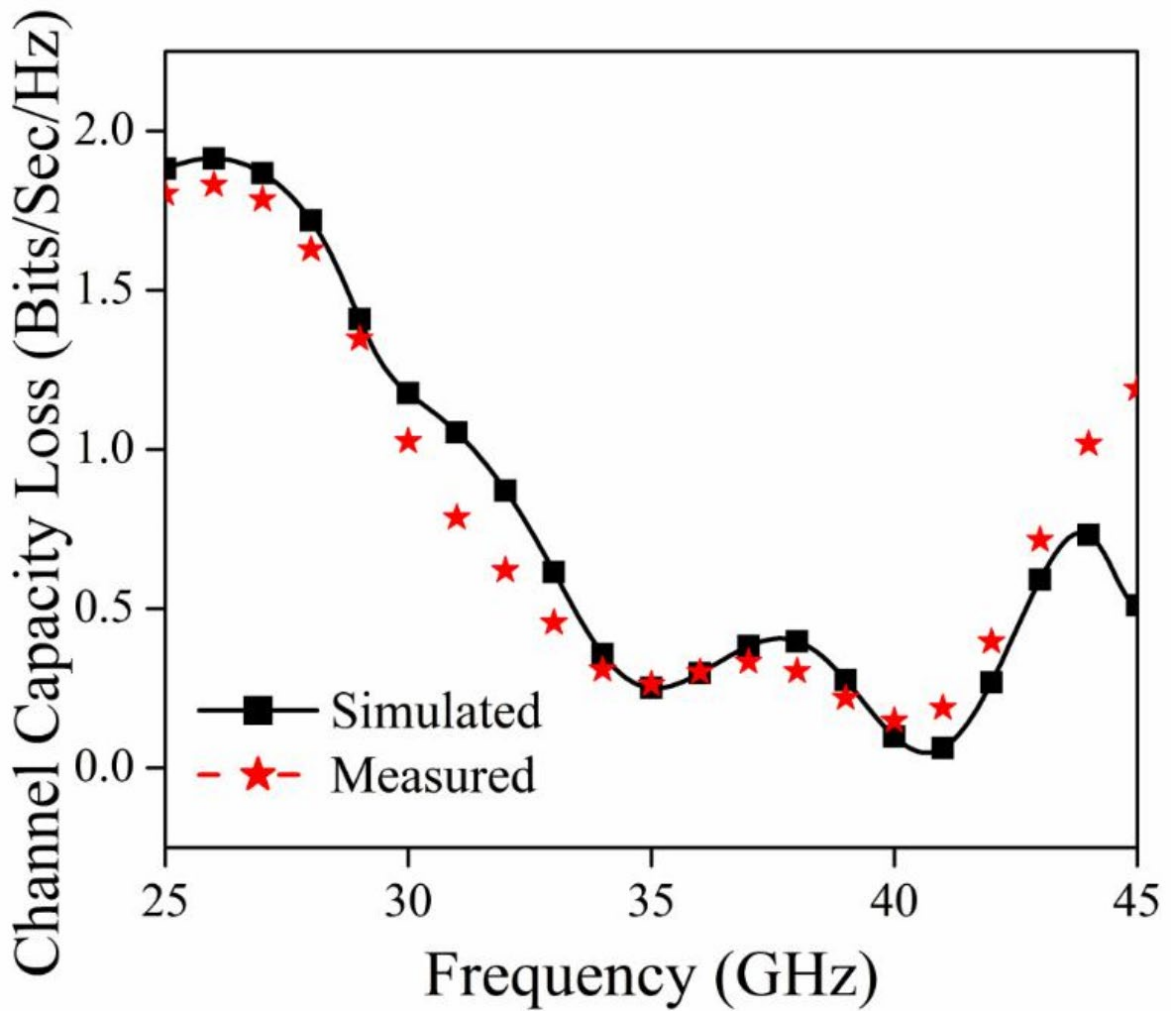
CCL graph for the proposed MIMO antenna module.

Furthermore, channel capacity loss (CCL) dictates the upper limit of information that may be sent across a wireless communication channel. The MIMO antenna system requires a CCL (Channel Capacity Limit) that is less than 0.5 bits per second per Hertz^37^. The equation used to compute the CCL may be found in Table 3. The calculated and observed values of CCL for the proposed MIMO antenna are below 0.5 bits/sec/Hz, as seen in Fig. 16 over the whole frequency spectrum.

**Fig. 17:**
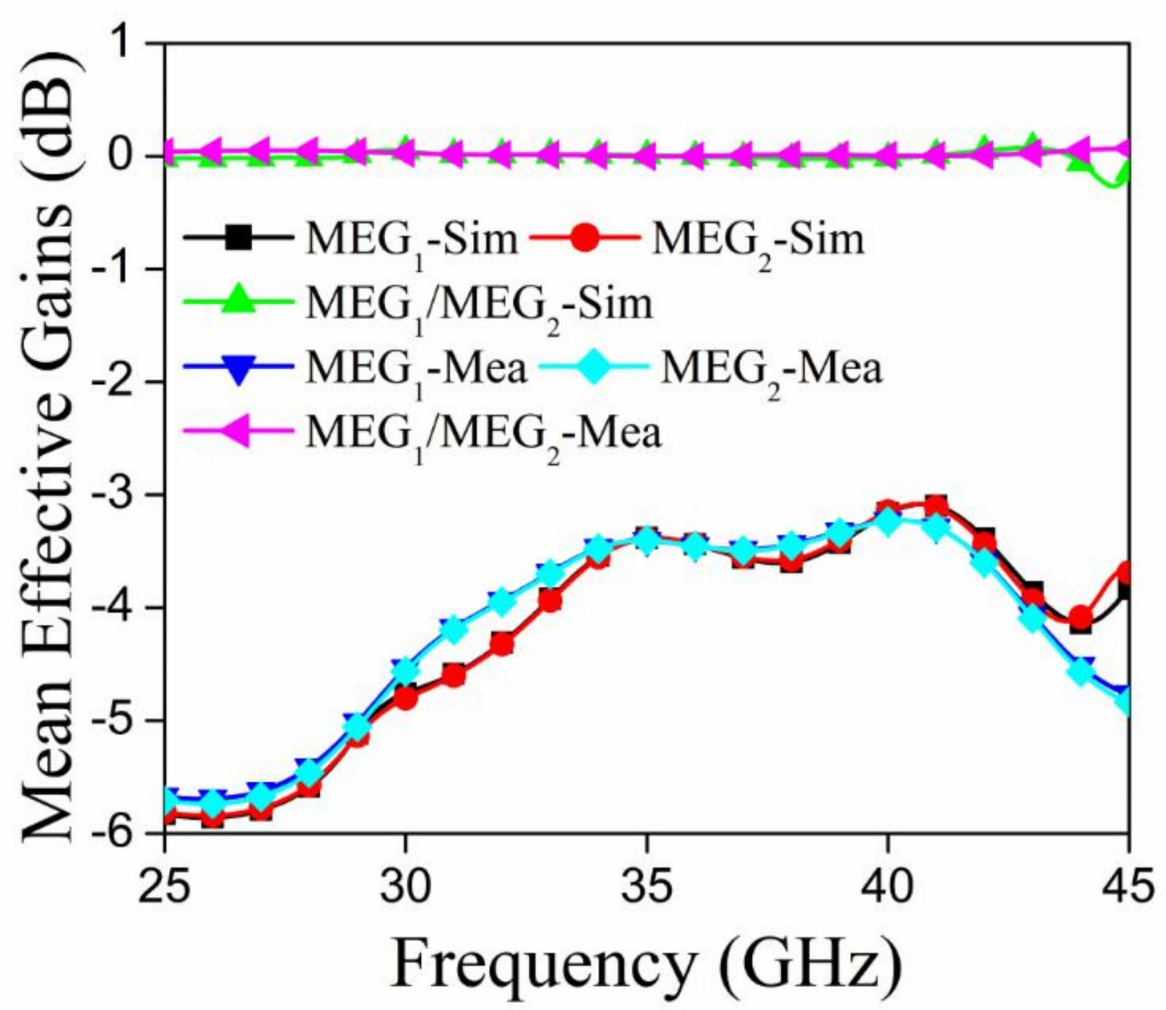
MEG graphs for the proposed MIMO antenna module.

An important diversity statistic for multiple-input multiple-output (MIMO) antennas is the mean effective gain (MEG), which is defined as the ratio of the received power of the MIMO antenna to that of the isotropic antenna. When the MEG1/MEG2 ratio is below 3 dB, the performance of a MIMO antenna is enhanced^38^. The value of the MEG may be determined using equations mentioned in Table 3. Figure 17 shows the MEG1, MEG2, and MEG1/MEG2 plots for the suggested radiator; the MEG1/MEG2 value is quite close to 0 dB.

### Novelty justification

To justify the novelty of the proposed module, a comparative table is introduced in Table 4 in which the comparative investigation is carried out between the previously reported metasurface-enabled MIMO antenna for mm-wave applications. According to Table 4 in the previously mentioned meta-antenna, the metasurface was not co-planar, resulting in increased isolation but also increased overall volumetric dimension and complexity of the construction. This study incorporates a conformal planar metasurface to improve the isolation, hence increasing its compatibility with printed antennas. Compared to all the reported antennas the proposed design has maximum B.W of 10 GHz in mm frequency region and the compact configuration.

**Table 4:** Comparative investigation of the proposed Module with other similar Meta surface enabled MIMO antenna for mm wave communication.

References	Methods	Complexity design	Area (mm^3^)	Impedance bandwidth (GHz)	Gain (dB)	Enhancement in isolation (dB)
^39^	Manipulating the surface currents of the modes	Linear	13 × 18 × 1.6	23–31	9.43	18
^40^	Metasurface Backed MIMO antenna	Linear	12.4 × 12.4 × 0.51	24.5–31	11	22.5
^41^	Square ring resonator (SQRR) metasurface enabled MIMO antenna	Linear	12 × 12 × 0.51	24.25–29.5	9.4	26
^42^	Metasurface as superstrate	Non-linear	20.5 × 20.6 × 0.508	27.38–33.34	12.7	21
^43^	Metasurface as superstrate	Non-linear	12 × 12 × 0.8	24.25–29.5	5.5	21
Proposed	Conformal meta surface absorber with MIMO antenna	Linear	12 × 10 × 0.8	32.5–42.5	5.5	26

## Conclusion

The proposed module represents a novel dual port metasurface absorber-enabled MIMO antenna for wideband millimeter wave communications for future-generation wireless devices. The exceptional bandwidth with compact configuration makes the proposed module ideal. The key features of the proposed work include the concept of optimizing the conformal metasurface absorber with the help of three important deep learning models (DNN, LSTM and Transformer) in order to achieve the maximum absorption which results in maximum possible isolation in the compact structure. All the findings of the proposed module are investigated and a comparative investigation is also carried out with meta meta-enabled MIMO antenna for mm-wave communications. The pertinent simulated results and their harmony with the measured findings make the proposed module a suitable contender for future wireless communication.

## Data Availability

The datasets used and/or analyzed during the current study available from the corresponding author on reasonable request.
